# Exploring the neurobiology of Merge at a basic level: insights from a novel artificial grammar paradigm

**DOI:** 10.3389/fpsyg.2023.1151518

**Published:** 2023-05-23

**Authors:** Yang Liu, Chenyang Gao, Peng Wang, Angela D. Friederici, Emiliano Zaccarella, Luyao Chen

**Affiliations:** ^1^Max Planck Partner Group, School of International Chinese Language Education, Beijing Normal University, Beijing, China; ^2^School of Global Education and Development, University of Chinese Academy of Social Sciences, Beijing, China; ^3^Method and Development Group (MEG and Cortical Networks), Max Planck Institute for Human Cognitive and Brain Sciences, Leipzig, Germany; ^4^Institute of Psychology, University of Greifswald, Greifswald, Germany; ^5^Institute of Psychology, University of Regensburg, Regensburg, Germany; ^6^Department of Neuropsychology, Max Planck Institute for Human Cognitive and Brain Sciences, Leipzig, Germany; ^7^Institute of Educational System Science, Beijing Normal University, Beijing, China

**Keywords:** Broca’s area, Merge, syntactic processing, natural language comprehension, fMRI

## Abstract

**Introduction:**

Human language allows us to generate an infinite number of linguistic expressions. It’s proposed that this competence is based on a binary syntactic operation, *Merge*, combining two elements to form a new constituent. An increasing number of recent studies have shifted from complex syntactic structures to two-word constructions to investigate the neural representation of this operation at the most basic level.

**Methods:**

This fMRI study aimed to develop a highly flexible artificial grammar paradigm for testing the neurobiology of human syntax at a basic level. During scanning, participants had to apply abstract syntactic rules to assess whether a given two-word artificial phrase could be further merged with a third word. To control for lower-level template-matching and working memory strategies, an additional non-mergeable word-list task was set up.

**Results:**

Behavioral data indicated that participants complied with the experiment. Whole brain and region of interest (ROI) analyses were performed under the contrast of “structure > word-list.” Whole brain analysis confirmed significant involvement of the posterior inferior frontal gyrus [pIFG, corresponding to Brodmann area (BA) 44]. Furthermore, both the signal intensity in Broca’s area and the behavioral performance showed significant correlations with natural language performance in the same participants. ROI analysis within the language atlas and anatomically defined Broca’s area revealed that only the pIFG was reliably activated.

**Discussion:**

Taken together, these results support the notion that Broca’s area, particularly BA 44, works as a combinatorial engine where words are merged together according to syntactic information. Furthermore, this study suggests that the present artificial grammar may serve as promising material for investigating the neurobiological basis of syntax, fostering future cross-species studies.

## 1. Introduction

The core of human language faculty (i.e., human language faculty in narrow sense) is a computational system (narrow syntax) that generates hierarchical syntactic structures, thereby being remarkably distinct from that of other living creatures (Hauser et al., 2002). Here we adopted “human language faculty” to include the present study in this framework, and to highlight the crucial capacity of hierarchical structure construction in humans for language comprehension and production (Indefrey et al., 2004; Hagoort, 2019) that go much beyond the sequential processing capacity of non-human animals (Hauser et al., 2002; Fitch and Hauser, 2004; Murphy, 2015; Berwick and Chomsky, 2016; Friederici, 2017, 2020; Goucha et al., 2017). Such competence is proposed to be based on a basic syntactic operation, Merge, which combines two elements (X and Y) together to form a *new* constituent (e.g., an X phrase, denoted as {_*XP*_ X Y}), and thus theoretically enables the generation of infinite hierarchical expressions (Chomsky, 1995; Miyagawa et al., 2013; Fujita, 2014; Zaccarella and Friederici, 2015; Berwick and Chomsky, 2016; Friederici, 2017, 2018; Hoshi, 2018, 2019). With regard to this minimal combinatory engine, a major question has arisen: What is the neural basis of Merge in human participants?

### 1.1. What is the neural basis of Merge in human participants?

Given the simplicity of Merge, a growing number of recent studies have shifted from complex to two-word syntactic structures to investigate the neural substrates of Merge at a comparatively basic level (Bemis and Pylkkänen, 2011, 2013; Westerlund and Pylkkänen, 2014; Westerlund et al., 2015; Zaccarella and Friederici, 2015; Zhang and Pylkkänen, 2015; Schell et al., 2017; Zaccarella et al., 2017b; Blanco-Elorrieta et al., 2018; Segaert et al., 2018; Fyshe et al., 2019; Maran et al., 2022). This shift occurs mainly because (a) compared with two-word phrases, sentences (especially syntactically complex ones) contain too many processing steps and confounding effects (such as propositional meaning, higher demand, and sentential context effect), which are difficult to disentangle (see also Pylkkänen, 2019; Maran et al., 2022), (b) two-word phrases are sufficient for the appreciation of the combinatory characteristics of language in both theoretical and experimental scenarios, and (c) findings and related paradigms using two-word phrases might be more compatible and illuminating for cross-population and cross-species comparisons (Maran et al., 2022). Nevertheless, a critical issue has emerged. What kind of two-word phrases could be utilized to investigate the neurobiology of Merge?

Efforts have been made to contrast two-word phrases composed of function and content words with non-combinable word lists (see Maran et al., 2022 for a recent review). In the seminal study of Zaccarella and Friederici (2015), a two-word phrase containing a German determiner and a pseudonoun [such as “Diese Flirk” (*This flirk*)] elicited significant activation of the ventral-anterior cluster of Brodmann area (BA) 44 within Broca’s area in the left hemisphere when compared to the word-list condition [e.g., “Apfel Flirk” (*apple flirk*)]. This Merge paradigm at the basic two-word level was different from the syntactic priming studies as well as the “red boat” paradigm (Pylkkänen, 2019, 2020) in exploring the neural substrates of Merge, thus purifying the syntactic processes. The result was further replicated by a series of follow-up studies in which function-content word pairs (i.e., mergeable phrases) were utilized (Schell et al., 2017; Wu et al., 2018). A recent meta-analysis (Zaccarella et al., 2017b) further converged on the notion that Broca’s area, especially the left BA 44, supports Merge as the core syntactic region.

However, in stark comparison, two-word phrases containing only content/open class words (such as adjective-noun phrases) used in magnetoencephalography (MEG) studies using the “red boat” paradigm presented distinct activation patterns (Bemis and Pylkkänen, 2011, 2013; Westerlund and Pylkkänen, 2014; Westerlund et al., 2015; Zhang and Pylkkänen, 2015; Neufeld et al., 2016; Pylkkänen, 2019, 2020; Fló et al., 2020). For instance, the comparison between the noun of the content-word phrase (e.g., “red *boat*”) with that of the word list (e.g., “xtp *boat*”) uncovered an earlier response in the left anterior temporal lobe (aTL), later followed by the peak of the left ventromedial prefrontal cortex (vmPFC) (Bemis and Pylkkänen, 2011, 2013). When the semantic specificity of the adjective/moderator had varied (such as “Asian food” vs. “Indian food”), the second noun would again elicit an early response in the left aTL (Westerlund and Pylkkänen, 2014; Zhang and Pylkkänen, 2015). These results indicated that the left aTL might play a central role in conceptual combination, a semantic mechanism theoretically different from Merge in the syntactic field. Schell et al. (2017) further identified the activation of the left BA 45 for selectively processing adjective-noun phrases and thus emphasized the syntactic profile of determiner-noun phrases. Therefore, studies using natural language materials disputed the word categories of the two-word structures, and an optimal two-word structure for highlighting syntactic processes seems to, at least, contain a function word, which might amplify the power to detect Merge while reducing the semantic interference (see also Maran et al., 2022).

### 1.2. Toward the artificial hierarchical syntactic structure-building grammar paradigm

**FIGURE 1:**
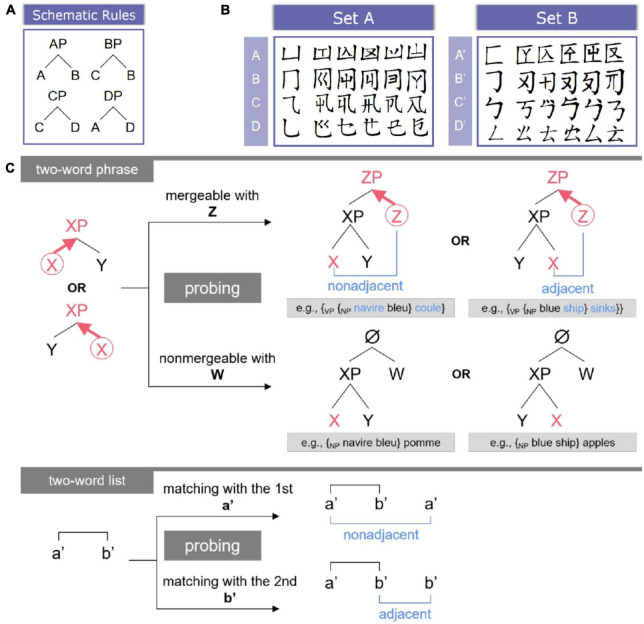
Experimental materials and tasks. **(A)** Schematic rules of HG. It is worth noting that these schematic rules as well as the categories were replaced by the materials from **(B)**. **(B)** Two sets of the pseudo-Chinese characters. **(C)** Task illustration. For two-word phrases, the Head category (highlighted by the red circle) would be detected, and it would project to (bold arrow) the higher syntactic level to label the whole phrase. The mergeable sub-condition could generate either non-adjacent or adjacent linear associations. Natural language examples (in either French or English) were also provided for the structure condition.

In order to exclude the semantic confounders and to guarantee the hierarchical nature of syntactic processing in a purer fashion, a novel artificial grammar previously called “hierarchical syntactic structure-building grammar” was created originally in Chen et al. (2021a). Here, the expression “syntactic structure” refers to the linguistic description of treating language strings as hierarchical structures built up on the basis of syntax, which is different from other structures such as thematic structures, event structures, and the like. For the convenience of understanding, we simplified the name of this grammar as “hierarchy building grammar” (HG). The HG contains structure-building rules applied to sets of functionally distinct categories (see Figure 1A). For instance, to process the sequence “ABD,” participants must merge category “A” with “B” first to form an A phrase (AP) “{_*AP*_ A B},” which syntactically belongs to category A, and thus {_*AP*_ A B} can be further merged with D to form a D phrase (DP) “{_*DP*_ {_*AP*_ A B} D}.” This process is ecologically compatible with human syntax (see also Figure 1C for natural language examples). Consider “the dog barks,” the determiner (“the”) and the noun (“dog”) will be merged as a determiner phrase (DetP) {_*DetP*_ the dog}, and it is further merged with the verb (“barks”) to form a verb phrase (VP) {_*VP*_ {_*DetP*_ the dog} barks}. Hence, HG ensures that for each instance of Merge, a higher syntactic node will be generated and itself will be recursively merged with other elements to form more complex syntactic hierarchies.

The HG has been utilized to generate four-word sequences whose complexity is much beyond that of basic two-word phrases, and the processing of these complex syntactic structures was contrasted with multilevel association sequence processing in the auditory modality (Chen et al., 2021a). The results showed that BA 44 and the left posterior temporal lobe (pTL) were highly activated and effectively connected for implementing Merge when participants heard the structured sequences. Nevertheless, the four-word HG complex structures were rather difficult to process, and as mentioned before, complex structures are “elephants” criticized for involving potential confounders, requiring comparable control conditions.

Therefore, for the first time, by generating minimal two-word phrases based on HG to compose the structure condition, the present study aims to explore Merge in a functional magnetic resonance imaging (fMRI) experiment in healthy adult humans. Accordingly, we developed a flexible *artificial grammar paradigm* in which participants must merge both words into a phrase to access the syntactic category of this phrase and to further judge its mergeability with the probing item. The probing item is crucial to ensure the merging of the previous two words. From this perspective of “two-word phrase plus one probing item” on the basis of HG, we call this whole process “Merge at a *basic* level,” and treated this as a novel paradigm in the present study. Moreover, a control condition using non-mergeable two-word lists (i.e., word-list condition) was also designed to control for other general cognitive effects (e.g., the working memory effect). In addition, materials were presented in the *visual* modality to test whether the processing of HG-based structures is modality-general (i.e., supramodal). By contrasting the structure condition with the word-list condition, we expected to detect the supramodal activation of Broca’s area (e.g., the left BA 44), the region proposed to be critical for core human language abilities.

Furthermore, natural language comprehension performance was measured using complex natural language sentences, simple sentences, and word lists composed of real words. These behavioral data would be correlated with the data acquired in the present artificial grammar paradigm, such as signal intensities of the related brain areas and the behavioral indices of processing the artificial structures, to directly assess the relationship between the process at work for the current artificial grammar paradigm and natural language performance.

If Broca’s area (especially, BA 44) could be activated similarly to the processing of natural language materials and if the data acquired from this artificial grammar paradigm show potential relationships to natural language comprehension performance, such an artificial grammar should be considered as ecologically valid for mimicking human syntax, and its basic-level processing paradigm should be sufficiently reliable for exploring the neurobiology of Merge.

## 2. Materials and methods

### 2.1. Participants

Twenty healthy adult native Chinese speakers (age: *M* = 22.65 years, SD = 1.84 years; 10 females) were recruited for this study. Their degree of right-handedness was confirmed by the Edinburgh handedness inventory (Oldfield, 1971). All the participants were late-bilinguals, non-musicians, with normal or corrected-to-normal vision, reporting no physical, psychiatric, or neuropsychological diseases. Informed consent was obtained in a manner approved by the Ethics Committee of Beijing Normal University, Beijing, China, and participants received remuneration after the completion of the whole experiment. No imaging data were excluded according to the criterion of excessive head motion artifacts (>2 mm in translation or >2° in rotation).

### 2.2. Materials

Given that the participants were native Chinese speakers, pseudo-Chinese characters were created for visual presentation (see Figure 1B). A major component stands for one category, and within the same category, five tokens were created by adding additional components or radicals to the major component, and each token (i.e., a pseudo-Chinese character) serves as one word. The rationales behind this procedure are as follows: (a) components of Chinese characters (especially, pictophonetic characters) are often able to reveal the syntactic/semantic categories of the corresponding characters (Wang, 1973; Tzeng and Hung, 1978; Feldman and Siok, 1999; Zhou and Marslen-Wilson, 1999; Chen and Yeh, 2015; Wang and Zhang, 2016; Yeh et al., 2017; Zhang et al., 2020), and (b) this procedure is comparable to the auditory materials from Chen et al. (2021a), in which each category was represented by a vowel, and word tokens were generated by combining the same vowel with various consonants. These pseudowords were totally unfamiliar to the participants, and the major components utilized in this experiment are unable to hint at the syntactic or semantic categories of the natural language (here, Mandarin Chinese). In case participants mixed the materials up to complete the tasks in the two conditions (i.e., the structure and the word-list conditions), two sets of pseudowords were created, with each one adopted for either the structure condition (Set A in Figure 1B) or the word-list condition (Set B in Figure 1B), and these two sets of materials were counterbalanced across participants.

According to the rules illustrated in Figure 1A, each time two categories could be merged, one of them should be the head, thus labeling the syntactic identity of the whole phrase. Merge is proposed to be independent of the internal word order (Chomsky, 2017, 2021; Goucha et al., 2017). Therefore, in this study, two mergeable categories (e.g., A and B) can be juxtaposed in a free-order fashion (i.e., “A B” and “B A” are both permitted). A total of 128 trials of two-word phrase tokens (i.e., the categorical phrases were expressed by the word tokens) were generated for the rule learning phase (see section “2.3. Procedures” for details), with 16 trials per block (8 blocks in total).

Moreover, in both the practicing and testing phases, a probing word followed each two-word phrase and required participants to judge whether it was mergeable with the previous phrase (see Figure 1C and section “2.3. Procedures” for details). Thus, if the probing word was considered, 16 trials were created for the practicing phase, with 8 trials per block (2 blocks in total), and 96 different trials, that is, trials with different word tokens to avoid superficial similarity effect (e.g., Opitz and Friederici, 2003, 2004; Opitz and Hofmann, 2015), were generated for the testing phase in the scanner, with 8 trials per block (12 blocks in total). Among these trials, half of the probing words were non-mergeable with the two-word phrases. Nevertheless, in line with Chen et al. (2021a), the underlying mechanism should be consistent. Participants should try to merge the probing word with the two-word phrase to check whether a higher syntactic node could be generated/labeled. Therefore, both of these stimuli were considered to reflect the hierarchical nature of syntactic processing. In addition, the positions and frequencies of each category (including the categories of the probing words) were carefully controlled (i.e., each category had the same frequency of its occurrence position) to ensure that participants were unable to develop alternative strategies to distinguish mergeable from non-mergeable stimuli. This finding means that each bigram (e.g., {{XY} Z} or {X {YZ}}), and the linear non-adjacent association (e.g., X—Z in XYZ), could equiprobably appear in both types of stimuli, making non-syntactic strategies such as the keyword strategy (including the edge effect), the (transition-) probability-based strategy, and the similarity-based strategy useless. Furthermore, according to the rules (Figure 1A), given the sufficient variability of the structure types (16 mergeable structures such as {{AB} B} and {{BA} D}, and 16 non-mergeable structures such as {{AB} C} and {{BA} A}) and the relatively limited response time (2 s) for each trial (see section “2.3. Procedures” for details), it should be rather uneconomical and inefficient to memorize these structure types without resorting to syntactic operations. In fact, this finding was also confirmed by the posttest interview, in which no participants reported using a memorization strategy.

Similarly, two-word lists were designed for the control condition, in which the word tokens were picked up from the other set of pseudowords (i.e., if Set A was used for the structure condition, Set B would be adopted for the word-list condition). For each trial, a probing word (e.g., a′) followed a two-word list (e.g., a′—b′), awaiting matching with its position of occurrence (e.g., whether a′ appeared at the first or second position) (see Figure 1C for illustration). This process canceled out the working memory (i.e., holding the two words in mind until resolving the matching task) and the repetition effects (i.e., the effects aroused by the repetition of the same items or templates in the trials such as a′—b′ a′ or a′—b′ b′), and the motion effects of button selection in the “structure > word list” contrast. The number of trials of the word-list condition was exactly the same as that of the structure condition in both the practicing and testing phases.

Since the pseudo-characters/words could appear in both structure and word-list conditions, the occasional semantic interpretation of these characters, if any, would be reduced under the contrast between the two conditions. Furthermore, 22 Chinese native speakers who did not participate in the experiment additionally rated the semantic plausibility (i.e., the degree of whether the materials were semantically plausible) of either single pseudo-characters/words or three-word structures (i.e., two-word phrase plus a probing mergeable/non-mergeable item/word, and all of these materials were used in the formal experiment) in a 5-point Likert scale (1: strongly implausible; 2: implausible; 3: unsure/neutral; 4: plausible; 5: strongly plausible). For the single characters, one-sample *t*-test against “2” showed that these pseudo-characters were highly implausible in semantics [*M* = 1.508, SD = 0.271, *t*(39) = −11.496, *p* < 0.001, Cohen’s *d* = −1.818]. As for the structures, similarly, the semantic plausibility of both mergeable and non-mergeable structures was significantly lower than “2” according to the separate one-sample *t*-tests [mergeable: *M* = 1.237, SD = 0.133; non-mergeable: *M* = 1.264, SD = 0.128; *t*s(95) −56.312, *p*s < 0.001, Cohen’s *d*s ≤ −5.747], and the paired-samples *t*-test result showed that there was no significant semantic plausibility difference between the mergeable and non-mergeable structures [*t*(95) = −1.650, *p* = 0.102, Cohen’s *d* = −0.168], indicating that both structures were highly semantically implausible. Therefore, the present stimuli were quite unlikely for the participants to process the structures via semantic strategies because of the highly low degree of semantic plausibility, and to judge the mergeability on the basis of meanings due to the fact that both mergeable and non-mergeable structures were comparably semantically implausible without significant differences, even when participants were explicitly encouraged to interpret the possible character/word and structure meanings.

Besides, it is noteworthy that the number of categories of the structure condition varies, when compared with that of the word-list condition. Nevertheless, participants had to merge the word categories together to judge the mergeability of the structures, which should go much beyond the mere processing of the number of word categories (see also section “4. Discussion”).

The natural language test adopted (a) Chinese complex sentences with relative clauses embedded, (b) simple sentences (Coordinated sentences, Thibault et al., 2021), and (c) word lists composed of real Chinese nouns or verbs. More details can be found in section “1. Natural language processing experiment” in Supplementary material.

### 2.3. Procedures

**FIGURE 2:**
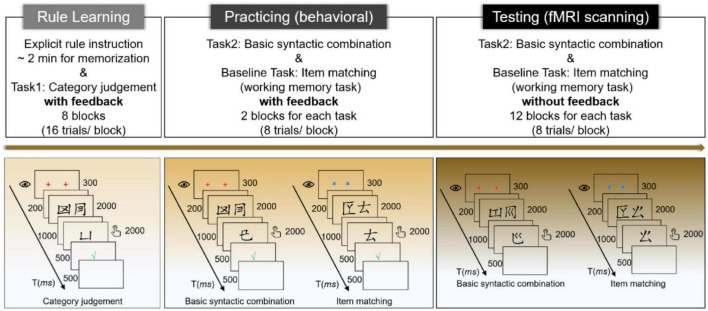
Experimental procedures.

As shown in Figure 2, this study had two stages, a behavioral stage prior to scanning and a testing stage in the scanner. At the very beginning, participants received *explicit* instructions on the HG rules as schematically depicted in Figure 1A, and they had two minutes to memorize these four rules. Then, participants underwent the 8-block rule learning phase, which aimed to consolidate their rule knowledge and required them to judge whether the probing category (represented by the major component of the characters) matched the category of the target two-word phrase (see also Chen et al., 2021a). For each trial, a screen of fixations (two red “+”) lasted for 300 ms to catch attention, followed by a blank screen (200 ms), and then the two-word phrase was visually presented for 2,000 ms, with a 1,000 ms blank screen at the end. The probing category (or word in other phases) appeared for 2,000 ms, during which participants were asked to respond by pressing the buttons with their index fingers. The correspondence between buttons and answers, and the response hands, were counterbalanced across participants. The response screen did not terminate until 2,000 ms was out and was followed by feedback (500 ms) to tune the learning performance. The intertrial interval (ITI) was set to 500 ms.

Before scanning, participants entered into the practicing phase, in which they were required to judge whether the probing word was mergeable with the target two-word phrase in the structure condition (we named the task *basic syntactic combination*) or which position the probing word appeared with regard to the previous two-word list in the word-list condition [called *item matching (working memory)* task here], as mentioned above. Each condition contained 2 blocks, and all four blocks were pseudorandomly mixed for each participant. The timing parameters of the presentation for each trial were the same as those of the rule learning phase. Nevertheless, the fixations for the word-list condition were two blue asterisks (“*”) to help participants differentiate and adapt to the target block efficiently (see also Matchin et al., 2017). The probing categories were replaced by the probing words. The types of fixations were counterbalanced across the conditions.

For the testing phase in the scanner, this study adopted a blocked design due to its comparatively higher detection power (Huettel et al., 2004). Two scanning sessions were set, each having 12 blocks (each condition had 6 blocks, and these blocks were pseudorandomized with an interblock interval of 24 s), and there was a 60-s rest between the two sessions. The presentation of each testing trial was the same as that of the practicing phase, except that no feedback was provided. After scanning, a structured post-test interview was conducted. All the experimental materials were visually presented by E-prime 2.0 (Psychology Software Tools, Inc., Pittsburgh, PA, USA).^1^ The whole experiment (including both behavioral and scanning stages) lasted for approximately 1.5 h.

The same participants were invited back to undergo the behavioral natural language comprehension experiment after one semester (approximately 6 months). For both complex and simple sentence conditions, participants were asked to judge the probing sentences, whereas for the word-list condition, they were required to judge whether the target word appeared at the probing position (i.e., whether a word appeared at a given position) (see section “1. Natural language processing experiment” in Supplementary material for more details).

### 2.4. Behavioral data analyses

Accuracy for each phase was tested against the random level (50%) by one-sample *t*-test to ensure the learnability of the rules and participants’ ability to manage all of the conditions. Then, in the case of the trade-off effect between accuracy and reaction time (RT) for a correct response, these two behavioral indices were synthesized into one value (called *behavioral performance* hereafter) via the equation “RT × [1 + 2 × (1 − accuracy rate)],” and a lower value indicates better performance (i.e., higher accuracy with lower RT) (Lyons et al., 2014; Goffin and Ansari, 2016; Morsanyi et al., 2017). Two-way repeated-measures ANOVA with “phase (practicing vs. testing) × task (structure vs. word list)” was performed. Paired-sample *t*-tests were used for *post hoc* comparisons with Bonferroni-corrected *p*-values.

Moreover, for exploration, Spearman correlation tests were performed between the behavioral performances of all the phases (i.e., rule learning, practicing, and testing phases, also including the natural language comprehension performances) separately with *p*-values uncorrected. Statistical analyses were performed by R (version 4.2.2) and JASP (version 0.16.4).^2^

### 2.5. Imaging data acquisition

Magnetic resonance imaging data were acquired via a 3.0-Tesla Siemens PRISMA magnetic resonance scanner (Siemens AG, Erlangen, Germany) with a 64-radiofrequency-channel head coil.

A T2*-weighted gradient echo planar imaging (EPI) sequence was adopted with the following parameters for functional data acquisition: repetition time (TR) = 2,000 ms; echo time (TE) = 30 ms; flip angle (FA) = 90°; field of view (FOV) = 208 mm × 208 mm; base resolution = 104 mm × 104 mm; in-plane resolution = 2 mm × 2 mm; slice thickness = 2 mm; number of slices = 64; gap = 0 mm; and alignment to the AC-PC plane. Signals from different slices were acquired by the multiband scanning technique (multiband factor = 2) to efficiently minimize slice-timing effects.

High-resolution anatomical T1-weighted images for coregistration were acquired according to the following parameters: TR = 2,530 ms; TE = 2.27 ms; FA = 7°; FOV = 256 mm × 256 mm; base resolution = 256 mm × 256 mm; in-plane resolution = 1 mm × 1 mm; slice thickness = 1 mm; and number of slices = 208.

### 2.6. Imaging data preprocessing

The imaging data were preprocessed by DPARSF 5.1 Advanced Edition (DPARSF: Data Processing Assistant for Resting-State fMRI, Yan et al., 2016), implemented in MATLAB R2020b. The preprocessing steps followed Yan et al. (2013), including (a) removing the first 4 volumes to reduce the magnetic saturation effect, (b) slice time correction, (c) field mapping, (d) spatial realignment, (e) coregistration, (f) segmentation (New segment + DARTEL), (g) nuisance covariate regression (polynomial trend: 1, linear detrending) including head motion regression using the Friston-24 model, (h) normalization of the images to the echo planar imaging (EPI) template based on Montreal Neurological Institute (MNI) stereotactic space to minimize cerebral differences between participants and resampling the images into 2 mm × 2 mm × 2 mm, and (i) smoothing the images with a 3D Gaussian kernel with full-width at half-maximum (FWHM) of 4 mm.

### 2.7. Whole-brain level analyses

Whole-brain analyses were performed using SPM 12^3^ implemented in MATLAB R2020b. At the first level, a general linear model (GLM) was set up for each participant by adding the structure and word-list conditions as two regressors of interest, with the onset and duration (48 s) of each block modulated as a boxcar function, which was further convolved with a canonical hemodynamic response function (HRF). Subsequently, the data were high-pass filtered at 128 Hz to eliminate low-frequency drifts.

At the second level, we were particularly interested in the contrast between the structure and word-list conditions, and based on the individual “structure > word list” contrasts at the first level, a one-sample *t*-test [i.e., “(structure > word list) > implicit baseline (fixation)] was performed on the group data to test against the null hypothesis that there were no activation differences between the structure and word-list conditions. Following Yan et al. (2013), each individual’s mean framewise displacement (FD) Jenkinson value was modeled as a covariate to regress out the head motion artifacts at the group level. Following Woo et al. (2014) and Wu et al. (2018), the whole-brain activation results were reported with a cluster-level familywise error (FWE)-corrected threshold of *p* < 0.05 using the cluster defining uncorrected threshold at *p* < 0.001 at the voxel-level, cluster size (*K*_*E*_) ≥ 20. The activated clusters with the proportion of white matter <1/3 from the SPM(T) maps would be reported.

Of note, whole-brain activation of each condition [i.e., under the contrast of either “structure > implicit baseline (fixation)” or “word list > implicit baseline (fixation)”] was also analyzed via one-sample *t*-test to ensure normal processing before contrasting “structure” with “word list” (see section “2. Whole-brain level activation results for each condition” in Supplementary material for details).

### 2.8. Region of interest analyses

#### 2.8.1. Group-level ROI analyses

To specify the syntactic neural basis within the language network, a 220 participant-based functional left-hemispheric language atlas extended from Fedorenko et al. (2010)^4^ was adopted as the language mask for small volume correction (SVC) to identify the peak activity coordinates of the related language regions under the “structure > word list” contrast. We further checked whether the key semantic regions (i.e., regions supporting meaning composition) within this language atlas were functionally suppressed in the “implicit baseline (fixation) > (structure > word list)” contrast (see also Chen et al., 2021a).

Moreover, given that the IFG in this language atlas did not differentiate BA 45 from BA 44, whereas these two regions have been proposed to have functional, macroanatomical, and microreceptoarchitectonic differences (e.g., Amunts et al., 1999, 2010; Hagoort, 2005; Grodzinsky and Friederici, 2006; Vigneau et al., 2006; Tyler et al., 2010; Fedorenko et al., 2011; Friederici, 2011, 2017; Pallier et al., 2011; Hagoort and Indefrey, 2014; Goucha and Friederici, 2015; Matchin et al., 2017), the anatomical left BA 44 and BA 45 masks (Amunts et al., 1999) were extracted from the maximum probabilistic cytoarchitectonic maps of the SPM Anatomy Toolbox 2.2b (Eickhoff et al., 2005) to compose Broca’s area (see also Zilles and Amunts, 2018), and it was applied to the SVC analysis of “structure > word list” to further specify the peak activity within the left IFG at the group level. The significance threshold of the group-level region of interest (ROI) analyses was defined as: a cluster-level familywise error (FWE)-corrected threshold of *p* < 0.05 using the cluster defining uncorrected threshold at *p* < 0.001 (voxel-level), cluster size (*K*_*E*_) ≥ 20, and the proportion of white matter <1/3.

#### 2.8.2. Individual-level ROI analyses: a functional localization approach

Concerns about the limitations of group-level activation analyses (especially, for Broca’s area) have been recently raised due to factors such as individual brain structural variability, the sensitivity of group-averaged signal detection, and the functional resolution of distinct regions, thus hampering the interpretation of human cognitive architecture (Gorgolewski et al., 2013; Fedorenko and Blank, 2020; Fedorenko, 2021). Hence, to evaluate the individual variability of the group-level activation results and, therefore, the present study further adopted a functional localization approach (see also Fedorenko et al., 2010; Blank et al., 2014; Malik-Moraleda et al., 2022). We performed individual-level ROI analyses within both the left IFG from the functional language atlas and the anatomical mask of Broca’s area (composed of BA 44 and BA 45), and for each participant, localizer-responsive voxels based on the *t*-values for the “structure > word-list” contrast were extracted as individual functional ROIs (fROIs). Subsequently, these individual fROIs within one mask (either IFG or Broca’s area) were synthesized as the group-level fROI, in which all the voxels were significantly activated for each participant. It is noteworthy that in the present study, functional localization was performed to evaluate whether the core syntactic regions (especially, BA 44) would be activated consistently across individuals with or without the threshold of picking up the top 10% most-responsive voxels, and whether the group-level ROI analysis result was robust across participants. We just hoped to assess the activation consistency across the participants, but this should not be treated as a replicate analysis due to the fact that the *same* data were used. More importantly, we wanted to correlate the functional activity with the behavioral performances in a more sensitive fashion. Signals extracted from the ROIs defined by the group-level ROI analyses might be suboptimal for the subsequent correlation tests, because of the defects of group-level activation analyses as aforementioned. Similar to Matchin et al. (2017), the individual-level ROI analyses aimed to identify the peak activity coordinates with more statistic power by enhancing the sensitivity of detecting the functionally relevant voxels under the contrast of “structure > word-list” at the individual level, thus beneficial to identifying the potential correlations between the brain signals and the task behavioral performances.

The averaged peak activity coordinates of the overlapped activation of the two group-level fROIs, if any, would be taken as the specific center for building up a 4-mm-radius sphere, whose time-series data would be extracted to calculate the percentage of signal change (signal change%) as signal intensity via MarsBaR 0.44.^5^ Exploratory Spearman correlation tests were performed between the signal intensities of the fROI(s) and the behavioral performances to establish the potential neurobehavioral relationships. We also compared the “signal change % and behavioral indices” correlations between the individual- and group-level defined ROIs so as to evaluate the robustness of the functional localization approach for exploring the correlative relationships between neural signals and behavioral performances (see section “4. Comparisons between different ROIs” in Supplementary material).

## 3. Results

### 3.1. Behavioral results

Descriptive statistics are summarized in Table 1. The accuracy of all the phases for each condition surpassed the random level [5.976 ≤ *ts*(19) ≤ 87.330, *p_*Bonf*_s* < 0.05, 1.336 ≤ Cohen’s *d* ≤ 14.713]. Given that the accuracy of both rule learning and testing phases was approximately 90%, the behavioral data suggested that the HG rules could be well acquired and successfully applied to the basic syntactic combination task.

**TABLE 1:** Descriptive statistics of behavioral data.

Condition	Accuracy			RT (ms)			Synthesized performance (ms)		
	**Mean**	**SD**	**SE**	**Mean**	**SD**	**SE**	**Mean**	**SD**	**SE**
**Structure**									
Rule	0.891	0.061	0.014	843.110	155.418	34.753	1,037.6	270.6	60.50
Prac	0.708	0.156	0.035	1,263.581	180.710	40.408	2,039.6	627.2	140.25
Test	0.909	0.053	0.012	1,065.457	163.868	36.642	1,263.8	251.4	56.22
**Word list**									
Prac	0.969	0.070	0.016	660.862	132.426	29.611	711.5	232.2	51.92
Test	0.977	0.043	0.010	630.772	107.184	23.967	662.4	142.0	31.75

Rule, rule learning phase; prac, practicing phase; test, testing phase.

**FIGURE 3:**
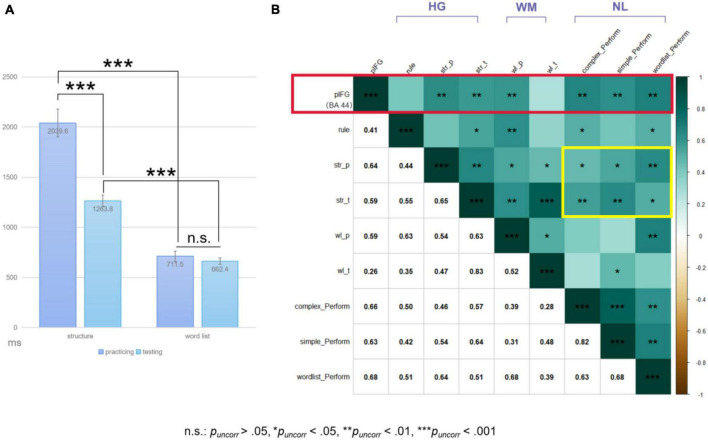
**(A)** Result plots of repeated measures ANOVA. The error bar stands for the standard error of the mean. **(B)** Spearman correlation test results (*rhos* were provided in the lower left quarter). HG, phases within the HG paradigm for the structure condition; WM, phases of the working memory task; NL, natural language conditions; str, the structure condition; wl, the word-list condition; p, practicing phase; t, testing phase. The red box highlighted the correlations between the signal intensity of the left pIFG and the performances of the structure condition at the testing phase and of the complex sentence processing performance. The yellow box highlighted the correlations between the structure condition performances and the natural language comprehension performances. To note, pIFG here was defined via the functional localization approach from the individual-level ROI analyses (see section “3.3.2. Individual-level ROI analysis results”).

The repeated-measures ANOVA on behavioral performance revealed significant interaction effects between phase and task [*F*(1,19) = 49.25, *p* < 0.001, $\eta_{p}^{2}=0.722$] and main effects of either phase or task [phase: *F*(1,19) = 43.51, *p* < 0.001, $\eta_{p}^{2}=0.696$; task: *F*(1,19) = 182.57, *p* < 0.001, $\eta_{p}^{2}=0.906$] (see Figure 3A). Paired-sample *t*-tests further identified significant performance changes in the structure condition from the practicing to testing phase [*t*(19) = 7.103, *p_*Bonf*_s* < 0.05, Cohen’s *d* = 1.588]. No significant performance changes could be found for the word-list condition [*t*(19) = 1.390, *p_*uncorr*_* = 0.181, Cohen’s *d* = 0.311]. Moreover, at each phase, the word-list condition showed better performance than the structure condition [practicing: *t*(19) = 11.077, *p_*Bonf*_s* < 0.05, Cohen’s *d* = 2.477; testing: *t*(19) = 17.493, *p_*Bonf*_s* < 0.05, Cohen’s *d* = 3.912]. As mentioned above, there were two scanning sessions, and the performance in the second session was much better than that in the first session [*t*(19) = −5.460, *p* < 0.001, Cohen’s *d* = −1.221). The performance difference (denoted as diff_*S*1 > *S*2_) between the two sessions was calculated for the structure condition and was compared with the performance difference (denoted as diff_*str* > *wl*_) between the structure and word-list conditions in the second session. Paired-sample *t*-test results showed that there was no significant difference between diff_*S*1 > *S*2_ and diff_*str* > *wl*_ [*t*(19) = −1.049, *p* = 0.307, Cohen’s *d* = −0.235].

The exploratory Spearman correlation test results are summarized in Figure 3B. As expected, the testing performance of the structure condition was correlated with the natural sentence comprehension performances (with complex: *rho* = 0.57, *p*_*uncorr*_ < 0.01; with simple: *rho* = 0.64, *p*_*uncorr*_ < 0.005).

### 3.2. Whole-brain level analysis results

**FIGURE 4:**
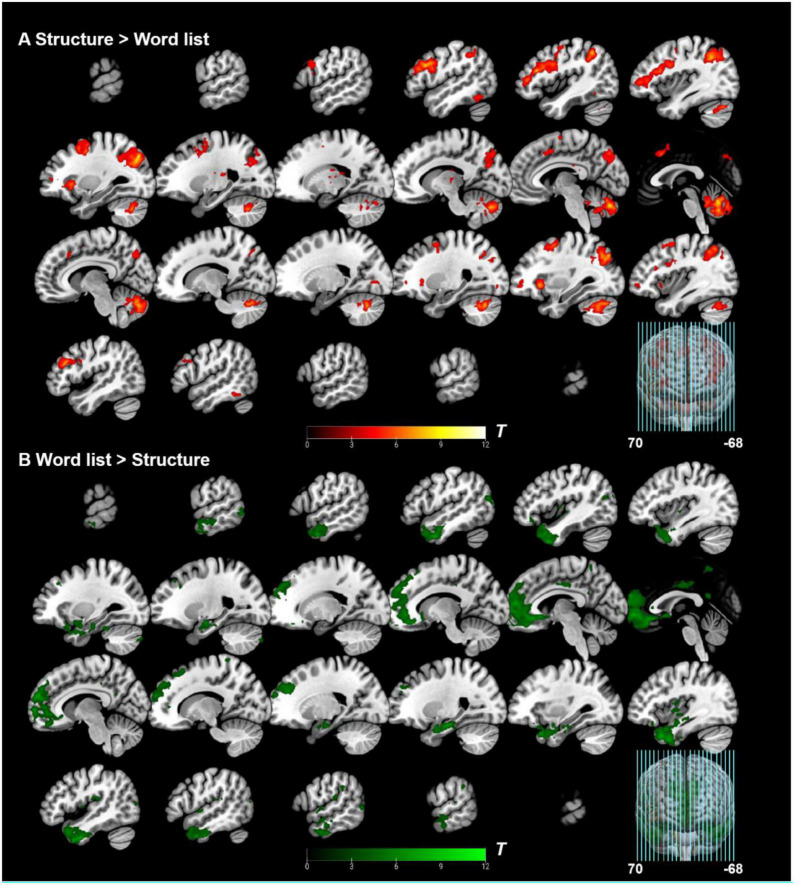
Whole-brain level analysis results. **(A)** Whole-brain level analysis under the contrast of “structure > word list”. **(B)** Whole-brain level analysis under the contrast of “word list > structure.” Activation thresholds: cluster-level: *p*_*FWE*_ < 0.05, voxel-level (cluster-defining): *p*_*uncorr*_ < 0.001, *K*_*E*_ ≥ 20.

The “structure > word list” contrast elicited significant activation of left IFG, left supplementary motor area (SMA), bilateral middle frontal gyrus (MFG), bilateral middle occipital gyrus (MOG), bilateral inferior temporal gyrus (ITG), and left thalamus at the whole-brain level. The reverse contrast “word list > structure” elicited significant activation of bilateral temporal pole (TP), bilateral MOG, left medial frontal cortex (mFC), right insula (Ins), right parietal operculum (PO), right middle temporal gyrus (MTG), bilateral precuneus (PCu), and right supramarginal gyrus (SMG). See Table 2 and Figure 4 for more information. In addition, both conditions were confirmed to be processed appropriately according to the single condition activation results (see Supplementary Table 1).

**TABLE 2:** Activation results at the whole-brain level and within the language ROIs.

Contrast	Region	*K*_*E*_	MNI peak coordinates (mm)			*t*-Value
			***x***	***y***	***z***	
**Whole-brain level**						
Structure > word list	Left MOG/AnG	2,070	−30	−76	38	10.60
	Right MOG	810	34	−76	40	8.74
	Left IFG/MFG	2,276	−50	18	28	8.58
	Right MFG	322	44	28	26	8.47
		325	36	14	54	6.84
		103	36	54	−2	5.95
	Left SMA	274	0	18	42	6.49
	Right ITG	60	54	−46	−20	5.96
	Left ITG	80	−50	−54	−11	5.10
	Left thalamus	41	−18	−14	2	4.59
Word list > structure	Right TP	2,090	44	6	−42	10.11
	Left TP	1,996	−44	8	−44	8.92
	Left mFC	5,325	−2	54	−6	8.55
	Right MOG	75	48	−80	12	6.91
	Right aINS	222	42	−4	4	6.80
	Right PO	119	48	−26	18	6.48
	Left MOG	108	−50	−78	20	6.02
	Right MTG	241	60	−12	−22	5.75
	Right PCu	76	2	−52	22	5.67
	Left PCu	99	0	−50	62	5.66
	Right SMG	72	62	−40	34	5.02
**Language atlas**						
Structure > word list	Left IFG	179	−50	18	28	8.58
Word list > structure	Left aTL	402	−48	12	−32	6.67
	Left AnG	30	−44	−72	20	5.40
**Broca’s area**						
Structure > word list	Left BA 44	340	−50	14	30	8.34

MOG, middle occipital gyrus; AnG, angular gyrus; IFG, inferior frontal gyrus; MFG, middle frontal gyrus; ITG, inferior temporal gyrus; SMA, supplementary motor area; TP, temporal pole; mFC, medial frontal cortex; aINS, anterior insula; PO, parietal operculum; MTG, middle temporal gyrus; PCu, precuneus; SMG, supramarginal gyrus; aTL, anterior temporal lobe; BA, Brodmann Area; *K_E_*, cluster size. Activation thresholds: cluster-level: *p_FWE_* < 0.05, voxel-level (cluster-defining): *p_uncorr_* < 0.001, *K_E_* ≥ 20. Only clusters with % white matter <1/3 were reported. Activation results of cerebellum were of no interest and not reported here.

### 3.3. ROI analysis results

#### 3.3.1. Group-level ROI analysis results

**FIGURE 5:**
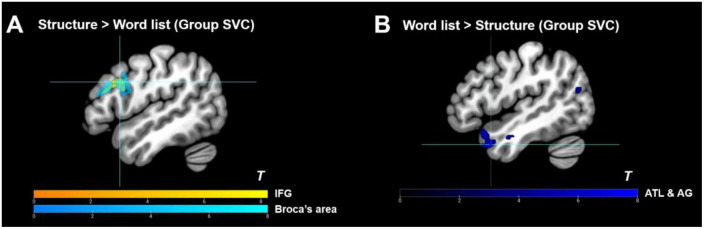
Group-level ROI analyses results. **(A)** Group-level ROI analyses (small volume correction) within Broca’s area and IFG of the functional language atlas under the contrast of “structure > word list”; **(B)** Deactivation within the functional language atlas under the contrast of “word list > structure.” IFG, inferior frontal gyrus; ATL, anterior temporal lobe; AG, angular gyrus. Activation thresholds: cluster-level: *p*_*FWE*_ < 0.05, voxel-level (cluster-defining): *p*_*uncorr*_ < 0.001, *K*_*E*_ ≥ 20.

As shown in Table 2, within the functional language atlas, only the left pIFG was significantly activated in the “structure > word list” contrast, and the semantic regions (e.g., Vigneau et al., 2006; Whitney et al., 2012; Davey et al., 2015; Hartwigsen, 2015; Jung and Lambon Ralph, 2016; Pylkkänen, 2019) were highly suppressed (see also Figure 5), thus further demonstrating the reliability of the current artificial grammar paradigm for disentangling syntactic areas from semantic areas (see also Chen et al., 2021a). Furthermore, to separate BA 44 from BA 45 within the IFG, an anatomical mask of Broca’s area was applied for the SVC, and the results showed that the peak activity was located in the left BA 44 at *x* = −50, *y* = 14, and *z* = 30 (Table 2 and Figure 5). Besides, we used single ROI masks extracted from the whole language atlas to perform SVC analyses under “structure > word list” separately, and found that except for IFG, the other regions (including the posterior temporal lobe) failed to show reliable activation [a cluster peaked at the left precentral gyrus was excluded because of a large amount of white matter activated, and the other regions did not research activation significance even at a liberal threshold (*p*_*uncorr*_ < 0.005, *K*_*E*_ = 0)].

#### 3.3.2. Individual-level ROI analysis results

**FIGURE 6:**
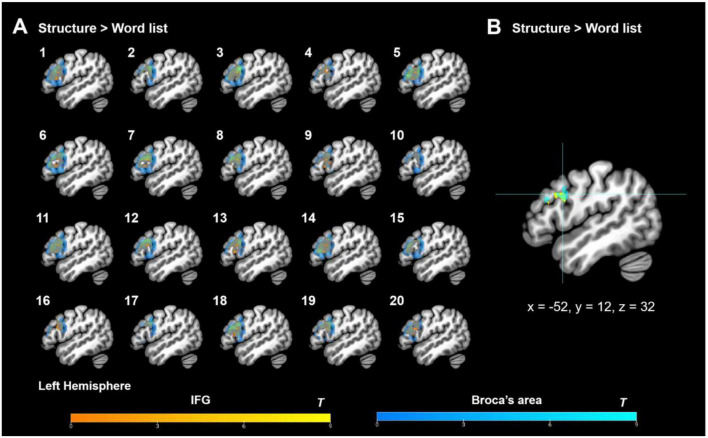
Individual-level ROI analysis results (without picking up the top 10% responsive voxels). **(A)** Individual fROIs for each participant (each number corresponds to one participant). **(B)** Group-level fROIs based on the individual data. Here IFG demotes to the IFG mask from the functional language atlas (http://web.mit.edu/evlab//funcloc/), and Broca’s area refers to the anatomical mask composed of the classic anatomical BA 44 (dorsally overlapping with parts of the inferior frontal sulcus and precentral gyrus) and BA 45 (Amunts et al., 1999, 2010; Zilles and Amunts, 2018; see also Eickhoff et al., 2005).

By assessing the congruence of the activation profiles across individual participants through the functional localization approach, the individual-level ROI analysis results showed that the individual fROIs of both IFG and Broca’s area were highly overlapped (see Figure 6A) and that the group-level fROIs converged on the left BA 44 at *x* = −52, *y* = 12, and *z* = 32 (Figure 6B), which shifted slightly (4 mm) from the peak activity coordinate (*x* = −50, *y* = 14, and *z* = 30) identified at the group level (see section “3.3.1. Group-level ROI analysis results”) and more slightly (∼3.46 mm) from the peak activity coordinate (*x* = −54, *y* = 14, and *z* = 30) when the *top 10%* localizer-responsive voxels were picked up in a stringent manner, thus providing complementary evidence to support the notion that the left BA 44 was robustly activated for syntactic processing (Merge) based on the HG rules in the present artificial grammar paradigm. More crucially, the individual-level ROI analyses identified almost the same peak activity coordinates with and without thresholding the top 10% most-responsive voxels, thus providing the subsequent signal change % analyses as well as the “signal change % and behavioral indices” correlation tests with the “anchored” coordinates for building up the ROIs. Here, we averaged the coordinates of the two fROIs (with and without the “top 10%” threshold), and got the mean peak activity coordinate: *x* = −53, *y* = 13, and *z* = 31, centered on which a 4 mm-radius sphere was built, and its signal intensity was calculated.

The Spearman correlation test results can be found in Figure 3B. It is intriguing that the signal intensity of pIFG (i.e., BA 44 here) was mainly correlated with the performance of the structure condition at both testing and practicing phases during scanning and with the processing performance of the natural language materials (practicing: *rho* = 0.64, testing: *rho* = 0.59, complex: *rho* = 0.66, simple: *rho* = 0.63, wordlist: *rho* = 0.68; *p_*uncorr*_s* < 0.01). Comparisons of the “signal change % and behavioral indices” correlations between the individual- and group-level defined ROIs could found in section “4. Comparisons between different ROIs” in Supplementary material, which would further demonstrate the necessity of adopting the functional localization approach for ROI analyses in the present study.

Furthermore, partial Spearman correlation tests were performed, and found that there was a significant trend of correlation between the signal intensity of pIFG and the complex sentence processing performance (*rho* = 0.455, *p* = 0.066) with the verbal working memory effects of the word-list processing eliminated. On the contrary, the signal intensity of pIFG did not correlate with the word-list performance when the artificial syntactic structure as well as the natural complex sentence processing performances were controlled for (*rho* = 0.278, *p* = 0.281). The partial correlation test results indicated that the activation of pIFG/BA 44 might be, to some extent, independent of the verbal working memory effects.

## 4. Discussion

The research aim of this study was twofold: (a) to specify the neural correlates of Merge, the basic syntactic combinatorial operation, in a newly developed artificial grammar paradigm to avoid the interference of semantic confounders in natural languages and (b) to demonstrate the ecological validity of this new artificial grammar paradigm concerning the degree of mimicking natural language processing (especially, syntactic processing on the basis of Merge).

Before assessing the neuroimaging results, relative concerns about the natural syntactic processing underlying the artificial grammar testing task are discussed. Considering that the stimulus materials were part of an artificial grammar that the participants had not learned in advance, we asked the participants to undergo the rule learning phase in order to ensure their familiarity with the artificial grammar. Given that the accuracy of the rule learning phase was approximately 90%, the behavioral data suggested that the HG rules had been well acquired. During the testing phase, the task was different from the learning phase. In the learning phase: the participants were only required to judge the grammatical categories of the two-word phrases, while in the testing phase, the participants needed to judge whether the two-word phrase could merge with the probing item further. Behavioral results indicated that the structures were well processed (accuracy: *M* = 0.908, SD = 0.062). Since the testing task had not been trained before, it indeed reflected the real-time application of syntactic rules to build up syntactic hierarchies during scanning. Thus, the present paradigm even went beyond plenty of artificial grammar learning/processing studies which trained participants on the task conditions before the actual scanning (see Uddén and Männel, 2018 for a systematic review).

The results showed that Broca’s area, especially the left BA 44, mainly overlapping with the left pIFG, could be reliably activated for Merge at both group and individual levels in a consistent fashion and that the signal intensity of this region correlates with the behavioral performance on the basic syntactic combination task for the structure condition and the natural language comprehension condition. It is noteworthy that the functional localization approach used in the individual-level ROI analyses could ideally detect the peak activity coordinates for exploring the correlations between neural signals and behavioral performances more sensitively (see also section “4. Comparisons between different ROIs” in Supplementary material). Moreover, the behavioral performance in the structure condition also correlated with the outcomes of natural language comprehension. With regard to these correlation results, we were unaware of the previous artificial grammar learning/processing studies that correlated their findings with natural language comprehension outcomes. These findings demonstrated the ecological validity of the present artificial grammar paradigm based on the empirical data, in addition to the theoretical rationale.

Due to the abstractness of the word categories designed in the present artificial grammar paradigm, participants could not resort to the natural language information to cope with the syntactic task. Our imaging results were in line with studies using natural two-word phrases containing function words (Zaccarella and Friederici, 2015; Schell et al., 2017; Zaccarella et al., 2017a,b; Wu et al., 2018) and further indicated that two-word phrases composed of concrete content/open class words might confound syntactic and semantic processes (cf., the “red boat” paradigm of Pylkkänen, 2019).

The involvement of Broca’s area (especially, BA 44) in the left hemisphere, as observed in the present study, has been identified previously in numerous syntactic processing studies using natural languages (e.g., Stromswold et al., 1996; Caplan et al., 2000; Vigneau et al., 2006; Grodzinsky and Santi, 2008; Makuuchi et al., 2009; Feng et al., 2011; Allen et al., 2012; Ohta et al., 2013; Hagoort and Indefrey, 2014; Goucha and Friederici, 2015; Zaccarella and Friederici, 2015; Wu et al., 2018; Wang et al., 2021), thereby supporting the ecological validity of this artificial grammar paradigm (see also Petersson et al., 2012; Uddén and Männel, 2018). Moreover, given that the experimental materials were visually presented differently from Chen et al. (2021a), the activation of BA 44 in this study supports the supramodal role of the area in handling syntactic operations, independent of the nature of the linguistic materials (acoustic syllables, visual characters, and the like) (see also Uddén et al., 2022). Furthermore, according to the functional localization approach (Fedorenko et al., 2010; Blank et al., 2014; Fedorenko, 2021; Malik-Moraleda et al., 2022), the involvement of BA 44 was highly consistent across the participants.

The peak activity detected in this study was dorsal to the one reported by Zaccarella and Friederici (2015) in the first two-word Merge paradigm. In addition to the localization variability caused by methodological factors such as imaging protocols or participants, a more critical difference comes from the syntactic task used in this artificial grammar paradigm. Rather than explicitly judging whether the two words could form a phrase (Zaccarella and Friederici, 2015, in the current experiment participants were required to tell whether the probing item could be merged with the former two-word phrase. Such a setting could avoid access to meta-linguistic knowledge (i.e., consciously knowing what a phrase means in linguistics) and amplify the Merge effects of hierarchical syntactic structure construction. That is, participants should merge twice. Meanwhile, they had to hold the newly merged two-word phrase in mind until merging with the probing item. Such a task as “holding embedded structures” was proposed to be a syntactically specific working memory, distinct from the verbal working memory associated with the linear distances of the dependencies, and was housed in the left inferior frontal sulcus (IFS) located dorsally to the IFG (Makuuchi et al., 2009, 2013). Therefore, the multilevel Merge might cause the activation peak of BA 44 to shift dorsally. This finding was also evidenced by the (partial) correlation results as reported in section “3.3.2. Individual-level ROI analysis results,” indicating that Broca’s area (especially, BA 44/pIFG) should play a syntactic role in language processing rather than acting as a mere working memory hub (Makuuchi et al., 2009, 2013; Rogalsky and Hickok, 2011; Goucha and Friederici, 2015; Zaccarella and Friederici, 2015; Zaccarella et al., 2017a,b).

There are a number of possible domain general processing strategies that should be discussed in the context of the present results. Because the structure condition showed significantly worse performance than the word-list condition, the involvement of Broca’s area (especially, BA 44) might be accounted for by a general cognitive control demand argument, as certain recent research has claimed (Fedorenko and Blank, 2020). On the one hand, previous studies proposed that Broca’s area did not respond to task difficulty (e.g., Friederici et al., 2006; Bahlmann et al., 2008). On the other hand, we found in our study that the performance in the second session was much better than that in the first session, and there was no significant difference between diff_S1 > S2_ and diff_str > wl_ (see section “3.1. Behavioral results” for more details). Thus, if Broca’s area could be activated due to the general cognitive loads as reflected by the performance differences, we would expect to detect its activation for both contrasts, “first session > second session” and “structure > word list,” whose performance differences were comparable (i.e., statistically the same). The results showed that “first session > second session” only elicited significant activation of the left insula, whereas “structure > word list” in the second session still activated Broca’s area (see also section “3. ‘Session 1 > Session 2’ vs. ‘structure > word list’ at the second session” in Supplementary material for more details). Therefore, the involvement of Broca’s area cannot be simply explained by general cognitive control demands.

Furthermore, the activation of BA 44 could not be ascribed to the mere template-matching strategy through syntactic priming. (a) From the perspective of experimental materials, different types of three-word syntactic structures (i.e., two-word phrase + one probing item) were created according to the HG rules, which included 16 mergeable and 16 non-mergeable types. The variability of the structure types was relatively large, and thus inefficient to be memorized as templates. This was further confirmed by the post-test interviews in which all the participants reported that they had built up hierarchical structures in mind. Moreover, in the stimuli pool (96 trials in total), only about 1/3 pairs of consecutive trials shared the same structure type. Therefore, the experimental materials had a complex diversity and it should not be ascribed to a mere priming effect among the materials. (b) From the perspective of procedures, participants in the rule learning phase only judged the categories of the two-word phrases, while in the practice phase of three-word phrases, there were only two blocks, thus making it unlikely to help participants solidify complete prefix grammar templates for eliciting priming effects before the testing phase. (c) From the perspective of data analyses, the existing priming studies focused on the priming-related activation suppression patterns (e.g., Sun et al., 2021), while in the present study, BOLD signals were averaged by synthesizing all the trials and mainly compared under the contrast of “structure > word-list,” instead of performing suppression analysis. Furthermore, pairs of word-list trials might also share the same linear structure/frame, and the priming effect, if any, would be subtracted under “structure > word-list” as well. Hence, based on these considerations, we believe that Merge was indeed under investigation in the present study rather than the mere priming effects of the prefixed grammar templates.

Finally, concerning the question of whether the current activation difference might simply be due to the number difference of the word categories between the structure and the word-list conditions we note the following: as mentioned in section “2.2. Materials,” participants were asked to judge the mergeability of the structures, which could not be solved by processing the word categories alone. The behavioral results indicated that participants could well differentiate mergeable and non-mergeable structures (see Table 1). Moreover, the present result that BA 44 was selectively activated for the structure condition was consistent with that of our previous study (Chen et al., 2021a), in which four-word complex structures built up on the basis of HG were compared with four-word linear sequences generated by the associative rules, and the number of word categories was the same between the two conditions. Therefore, instead of reflecting a mere “number-of-category effect” it should be the Merge operation on these word categories to build up syntactic hierarchies that elicited higher activation in BA 44.

In addition to activation in BA 44/pIFG, whole-brain results also revealed the activation of certain areas, which were quite consistent with the findings of Petersson et al. (2012) and would be discussed briefly. The MOG was activated bilaterally under the contrast of “structure > word list,” dorsal to the bilateral MOG identified under the “word list > structure” contrast. Several studies have identified MOG in reading, which was assumed to be responsible for orthographic processing, and showed greater activation in the pseudoword condition (e.g., Indefrey et al., 1997; Levy et al., 2009). Although pseudoword reading was required for both the structure and word-list conditions in the present study, under the structure condition participants might be more focused on word forms associated with different word category information for Merge, when compared with the word-list condition. Nevertheless, specific functional roles of the dorsal and ventral parts of MOG are yet unclear. Similarly, the ITG was also involved more in the structure condition. The peak activation of the left ITG was located in the visual word form area (VWFA), a region for visual word processing during reading (e.g., Dehaene et al., 2005; Glezer et al., 2009). Although the word-list condition also required to process the words, we reasoned that the “depth” of processing might be different between the two conditions. Participants just needed to memorize the appearances of the words to cope with the working memory task under the word-list condition, whereas the structure condition asked participants to access the detailed lexical information (especially, word category information), which even recruited the right ITG (possibly for a coarse analysis of the unfamiliar pseudo-words). Moreover, MFG also engaged in the structure processing bilaterally. This might be related to the higher executive-control demands of the structure condition when compared with the word-list condition (Yeo et al., 2011; Uddin et al., 2019; Chen et al., 2021a; Fedorenko, 2021). Also partially in consistence with Petersson et al. (2012), subcortical activation in basal ganglia, especially the left thalamus, was detected under the contrast of “structure > word list.” Basal ganglia was recently identified to be shared by both complex sentence comprehension as well as complex tool use (Thibault et al., 2021). In particular, thalamus was suggested to be a key region within the prefronto-subcortical network for cognitive control (Friederici, 2006). Thalamus along with the specific cortical activation of language processing might play a critical role in the online computation of combinatorial rules in word sequences (Wahl et al., 2008), and its connection to the prefrontal cortex responded to the complex conditions generally when the cognitive demands were high (Jeon et al., 2014). Lastly, the left SMC was further activated for the structure condition, which was previously proposed to support syntactic encoding and decoding (Indefrey et al., 2001; Haller et al., 2005; Snijders et al., 2009; Lee and Newman, 2010; Menenti et al., 2011), and might be involved in sequencing the syntactic structures (Segaert et al., 2012). To note, the reverse contrast of “word list > structure” was not of interest in the present study, and the activated regions might be related to verbal working memory capacity [such as SMG (e.g., Deschamps et al., 2014)] or to other non-syntactic processes.

It should be mentioned that in the present study we did not observe activation in the pTL, whose engagement in syntactic processing had been reported by previous studies (e.g., Humphries et al., 2001; Santi and Grodzinsky, 2010; Bemis and Pylkkänen, 2011, 2013; Pallier et al., 2011; den Ouden et al., 2012; Westerlund and Pylkkänen, 2014; Matchin et al., 2017, 2019; Zaccarella et al., 2017a; Wu et al., 2018; Chen et al., 2021a,b; see also Zaccarella et al., 2017b for a meta-analysis). The role of the pTL in the processing of natural language materials may be related to the following reasons. On the one hand, the pTL was assumed to be in support of semantic and syntactic information integration during (a) complex syntactic structure processing (Bornkessel et al., 2005; Friederici et al., 2009; Friederici, 2011, 2017; den Ouden et al., 2012; Hagoort, 2013; Goucha et al., 2017; Zaccarella et al., 2017a), (b) when holistic (and/or propositional) semantic representations should be constructed (e.g., Bemis and Pylkkänen, 2011, 2013; Del Prato and Pylkkänen, 2014; Schell et al., 2017), and during (c) the integration of multiple sources of information, like thematic-role assignment (e.g., den Ouden et al., 2012) or semantic predictability (Obleser and Kotz, 2010), especially when the semantic information is interfered with syntactic complexity/difficulty (Cooke et al., 2002; Friederici and Kotz, 2003; Constable et al., 2004). On the other hand, syntactic complexity alone also can lead to activation of the pTL. Evidence came from the artificial grammar sequence or jabberwocky sentence processing studies, reflecting the role of syntactic processing in complex sequences (e.g., Friederici et al., 2006; Chen et al., 2021a,^2023^), but the present study focused on Merge at the most basic level, whose syntactic operations were far from complex and therefore did not reach the level of arousing posterior temporal lobe activation, even when SVC was performed with a single ROI of the posterior temporal lobe at a liberal threshold. The null activation result of the pTL in the present study is highly consistent with Zaccarella and Friederici (2015). The involvement of the pTL has not been systematically investigated at the most basic level (i.e., the two-word phrase level), leaving factors such as the syntactic simplicity of materials and input modality unspecified. We would like to leave these issues for future explorations.

In summary, the present study replicated the critical involvement of Broca’s area, especially the left BA 44, for Merge at the basic level by developing an artificial grammar paradigm. Both the signal intensity of Broca’s area and behavioral performance were correlated with natural language comprehension performance, further demonstrating the ecological validity of this paradigm.

## 5. Outlooks

Several aspects are worth considering before applying this artificial grammar paradigm in future studies. Whether non-human animals are able to build up syntactic hierarchies via Merge is still mysterious. Artificial grammars deprived of semantic information were designed to tackle human syntax-related themes mainly concerning the specialty and neurobiology of human syntactic competence and comparisons with non-human animal rule-based sequence learning abilities (Petersson et al., 2012; Milne et al., 2018; Uddén and Männel, 2018; Morgan-Short, 2020; Petkov and Ten Cate, 2020). However, previous artificial grammars, such as (a) AX*^n^*B grammar, in which A is non-adjacently associated with B with center-embedded intervening elements (e.g., Newport and Aslin, 2004; Murphy et al., 2008; Endress et al., 2010; Ravignani et al., 2013; Seki et al., 2013; van Heijningen et al., 2013; Lu and Vicario, 2014; Sonnweber et al., 2015; Spierings and ten Cate, 2016; Chen and Ten Cate, 2017; Milne et al., 2018; Mueller et al., 2018; Versace et al., 2019), and (b) A*^n^*B*^n^* grammar, which generates multilevel association structures such as “A_1_A_2_B_2_B_1_” with the internally associated pair “A_2_B_2_” center-embedded in the outer pair “A_1_B_1_” (Fitch and Hauser, 2004; van Heijningen et al., 2009; Abe and Watanabe, 2011; Rey et al., 2012; Stobbe et al., 2012; Jiang et al., 2018; Ferrigno et al., 2020), were criticized to induce non-rule strategies such as counting and repetition/symmetry detection to solve tasks (e.g., de Vries et al., 2008; Beckers et al., 2012; Fitch and Friederici, 2012; Stobbe et al., 2012; Berwick et al., 2013; Friederici, 2018; Petkov and Ten Cate, 2020). More crucially, these non-adjacent rules, generating structures containing either single- or multilevel associations, are not sufficient to capture the hierarchical nature of human language. This lack of sufficiency is because a mere association (e.g., X—Y) is unable to generate a syntactic node higher than XP in a well-merged phrase {_XP_ X Y} (Perruchet and Rey, 2005; Friederici et al., 2011; Jeon, 2014; Goucha et al., 2017; Chen et al., 2021a) and because the processor should infer the internal nodes of the syntactic hierarchy during the processing of a human language sequence (Uddén et al., 2020). Such a problem is more severe in the adjacent dependency rules, as in (AB)^n^ grammar, which could generate minimal adjacent two-word pairs such as “AB” without necessarily generating a hierarchical structure. A number of studies have shown that non-human animals are capable of mastering these rules (e.g., Fitch and Hauser, 2004; Gentner et al., 2006; Herbranson and Shimp, 2008; Saffran et al., 2008; van Heijningen et al., 2009; Endress et al., 2010; Stobbe et al., 2012; Wilson et al., 2013, 2015; Chen et al., 2015; Heimbauer et al., 2018; Milne et al., 2018). The underlying learning strategies of (AB)^n^ grammar might be ascribed to sensitivity to transitional probabilities (e.g., Saffran et al., 1996, 2008; Chen et al., 2015), fixed positional information (e.g., Endress et al., 2010; Chen et al., 2015), and superficial similarity between training and testing stimuli (e.g., Opitz and Friederici, 2003, 2004; Lieberman et al., 2004; Herbranson and Shimp, 2008; Saffran et al., 2008; Hauser et al., 2012; Wilson et al., 2013; Opitz and Hofmann, 2015), thus not building syntactic hierarchies.

Therefore, the HG-based paradigm in the present study might be applied to future cross-species studies to substantially deepen our understanding of the evolution of our brain hardware for human language. Nevertheless, the adult participants were explicitly instructed on the HG rules, and they were well aware of how to construct syntactic hierarchies with consciously acquired rule knowledge in a relatively short time period. As for non-human animals, how intensive the learning/training should be and whether the rules are learnable in an implicit manner are yet unknown.

Moreover, since this paradigm is flexible in using experimental materials, will non-language elements, such as music notes and mathematical numbers, elicit a similar activation pattern of BA 44? If so, these results may be compared with processing in various cognitive domains.

## Data availability statement

The raw data supporting the conclusions of this article will be made available by the authors, without undue reservation.

## Ethics statement

The studies involving human participants were reviewed and approved by the Ethics Committee of Beijing Normal University, Beijing, China. The patients/participants provided their written informed consent to participate in this study.

## Author contributions

LC, EZ, and AF came up with the original idea. LC, YL, CG, and PW contributed to the experiment design. YL and CG contributed to the data acquisition. LC, YL, and CG analyzed the data. LC, YL, and CG completed the first draft of this manuscript, which was further revised by PW, AF, and EZ. All authors participated in the discussion of the results and prepared the revised version of the manuscript for submission.

## Footnotes

1
https://support.pstnet.com

2
https://jasp-stats.org

3
https://www.fil.ion.ucl.ac.uk/spm/

4
http://web.mit.edu/evlab//funcloc/

5
https://sourceforge.net/projrcts/marsbar/
