# Supplementary material for: Exploring the neurobiology of Merge at a basic level: insights from a novel artificial grammar paradigm
Source: Front Psychol. 2023 May 23;14:1151518. doi: 10.3389/fpsyg.2023.1151518 (PMC10242141; doi:10.3389/fpsyg.2023.1151518)
Supplement: Supplementary file 1 [file Data_Sheet_1.docx]

Supporting Information

Exploring the neurobiology of Merge at a basic level: Insights from a novel artificial grammar paradigm

**Yang Liu^1†^, Chenyang Gao^2†^, Wang Peng^3, 4, 5^, Angela D. Friederici^6^, Emiliano Zaccarella^6^, Luyao Chen^1 6 7*^**

^1^ Max Planck partner Group, School of International Chinese Language Education, Beijing Normal University, Beijing, China

^2^ School of Global Education and Development, University of Chinese Academy of Social Science, Beijing, China

^3^ Method & Development Group (MEG and Cortical Networks), Max Planck Institute for Human Cognitive and Brain Sciences, Leipzig, Germany

^4^ Institute of Psychology, University of Greifswald, Greifswald, Germany

^5^ Institute of Psychology, University of Regensburg, Regensburg, Germany

^6^ Department of Neuropsychology, Max Planck Institute for Human Cognitive and Brain Sciences, Leipzig, Germany

^7^ Institute of Educational System Science, Beijing Normal University, Beijing, China

**† These authors contributed equally to the current work.**

*** Correspondence:**Dr. Luyao Chen
luyaochen@bnu.edu.cn

# Natural language processing experiment

## Methods

### Participants

Participants were the same as those in the fMRI study as described in the *main text*.

### Materials

Three conditions were set (see Figure S1A) for Mandarin Chinese natural language processing: (a) the complex sentence condition: sentences with relative clauses embedded were generated; (b) the simple sentence condition: simple SVO sentences were combined to form Chinese sentences in a chronicle style; (c) the word-list condition: either noun lists or verb lists were created. The naturalness of each condition was evaluated with a 5-point Likert scale (1: totally unnatural; 5: totally natural) by 48 native Chinese speakers. A nonparametric Kruskal‒Wallis test was performed to assess the naturalness differences among these conditions and detected significant differences [*χ^2^*(2) = 92.877, *p* <.001]. Post hoc pairwise comparisons revealed that both types of sentences were more natural than the word lists (*p_Bonf_*s <.05), and there was no naturalness difference between these two types of sentences (*p_Bonf_* = 1).

For the sentence conditions, both correct and incorrect probing sentences were designed, and for the word-list condition, correct and incorrect relations between the probing word and its target position were set. The three conditions shared the same pool of words. Each condition contained 72 trials, half of which were incorrect. Word frequencies and the occurrences of the single words and the word pairs (such as a bigram composed of a noun and a verb or of two nouns/verbs) were carefully controlled so that participants were unable to predict the reaction when reading each sequence (i.e., a sentence or a word list). Bigrams of nouns or verbs of the word lists were also checked to exclude the potentially mergeable pairs.

### Procedures

After a semester, the same participants were recalled and underwent this behavioral natural language processing experiment. Before the actual experiment, participants practiced 8 trials for each condition to adapt. As shown in Figure S1B, stimuli of the conditions were visually presented word-by-word in a pseudorandomized order. For each trial per condition, a specific fixation (such as a red cross) occurred at the center of the screen and lasted for 300 ms, followed by a 100-ms blank. Each word was presented for 500 ms with a 100-ms blank in-between every two words. Before the response screen presenting a probing sentence or a relation, a 100-ms blank occurred. The response screen lasted for at-most 2000 ms and would terminate as soon as the first response was made. Participants were required to judge the correctness of the probing sentences or relations with the index finger of each hand. The fixation types and the response hands were counterbalanced across the participants. The stimuli were presented electronically using E-prime 2.0 (Psychology Software Tools, Inc., Pittsburgh, PA, USA; https://support.pstnet.com). The whole experiment lasted for approximately 30 min.

## Behavioral results

As mentioned in the *Main Text*, accuracy and reaction time (RT) were synthesized into one value called *behavioral performance*. One-way repeated-measures ANOVAs were performed with a condition factor (complex sentence, simple sentence, and word list), and the results showed a significant effect of condition [*F*(1,19) = 38.91, *p* <.001, *η² _p_* = 0.672] (see also Figure S1C). Post hoc tests showed that processing the simple sentences was better than those of the other two conditions [*ts*(19) ≥ 7.398, *p_Bonf_s* <.001, Cohen’s *ds* ≥ 1.654] and that no performance difference could be found between the complex sentence and the word-list conditions [*t*(19) = 0.677, *p_Bonf_* = 1, Cohen’s *d* = 0.151]. With regard to the cognitive loads, the syntactic complexity of the complex sentence condition was thus cognitively comparable with the working memory costs of the word-list condition. The simple sentences had lower syntactic complexity and working memory demands, and therefore, as expected, showed the best processing performance among the three conditions.

**
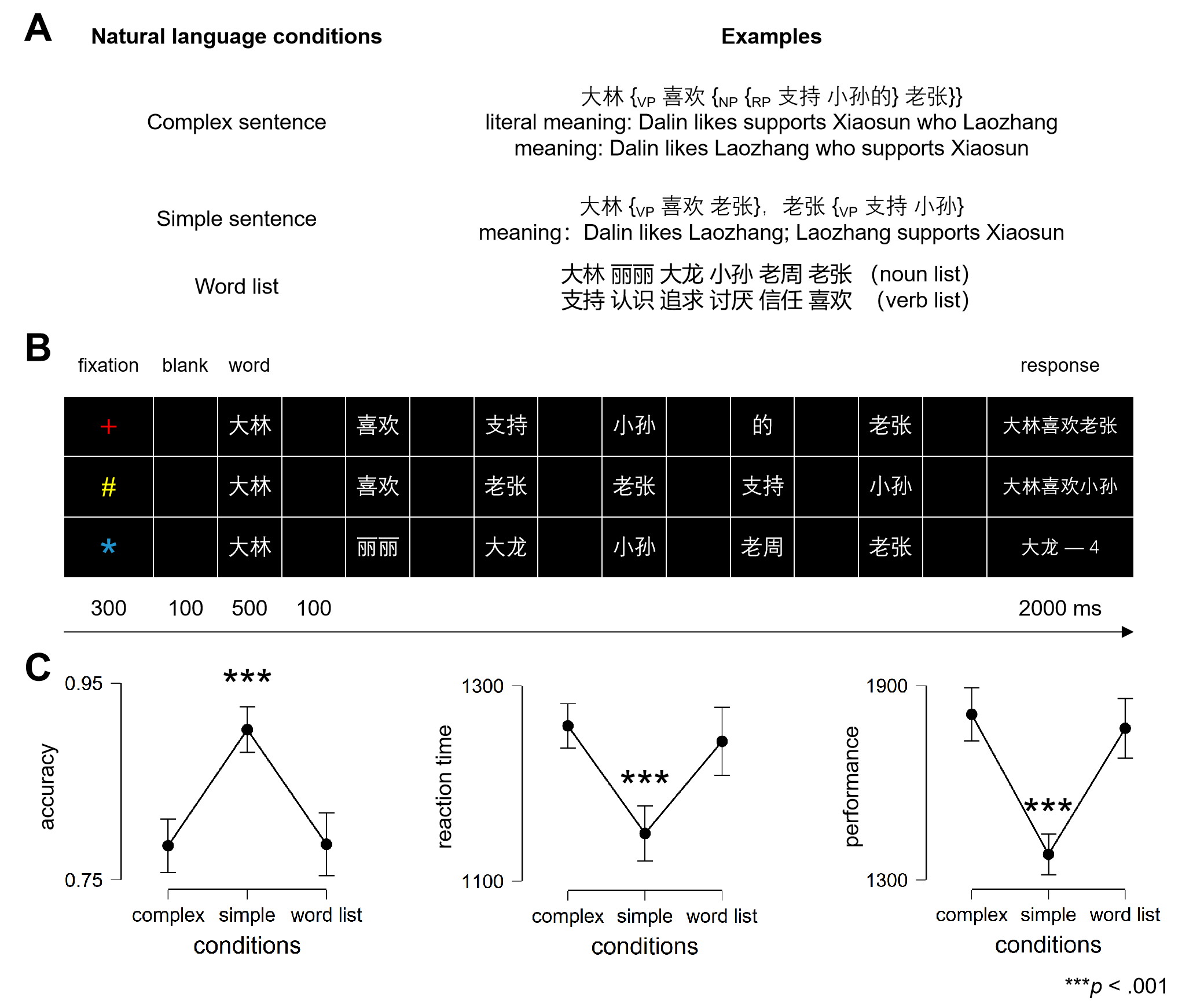
**

**Figure S1.** A: Natural language conditions and the corresponding examples. B: Procedures of the presentation of each trial for each condition. C: Plots of the behavioral results; each error bar stands for the standard error of the mean.

# Whole-brain level activation results for each condition

Whole-brain activation in each condition [i.e., under the contrast of either “structure > implicit baseline (fixation)” or “word list > implicit baseline (fixation)”] was analyzed via one-sample t-test to ensure normal processing of each condition. As shown in Table S1 and Figure S2, both conditions elicited reliable activation results, and these results could be further included in the contrasts between the two conditions.

**Table S1. Activation results for single condition at the whole-brain level**

| **Contrast** | **Region** | ***K_E_*** | **MNI Peak Coordinates (mm)** | | | ***T*-value** |
| --- | --- | --- | --- | --- | --- | --- |
|  |  |  | **x** | **y** | **z** |  |
| **structure > implicit baseline (fixation)** | | | | | | |
|  | Right SPL/ SOL | 1507 | 28 | -68 | 44 | 10.67 |
|  | Left IFG/ PreCG | 4593 | -46 | 4 | 30 | 10.66 |
|  | Left Ins | 381 | -32 | 18 | 2 | 10.05 |
|  | Right MFG | 174 | 50 | 30 | 30 | 5.47 |
| **Word list > implicit baseline (fixation)** | | | | | | |
|  | Right FG | 3047 | 44 | -64 | -20 | 10.84 |
|  | Left IOG | 3052 | -40 | -82 | -18 | 10.78 |
|  | Right SMC | 1079 | 2 | 4 | 52 | 8.02 |
|  | Right SPL | 427 | 28 | -68 | 44 | 6.23 |
|  | Right Putamen | 110 | 26 | 2 | -4 | 4.48 |

***Notes.*** Whole-brain level activation results for each condition. Activation results of cerebellum were of no interest and not reported here. Activation thresholds: cluster-level: *p_FWE_* <.05, voxel-level (cluster-defining): *p_uncorr_* < .001, *K_E_* ≥ 20. Only clusters with % white matter < 1/3 were reported. Abbreviations: SPL: superior parietal lobule; SOL: superior occipital lobe; IFG: inferior frontal gyrus; PreCG: precentral central gyrus; Ins: insula; MFG: middle frontal gyrus; FG: fusiform gyrus; IOG: inferior occipital gyrus; SMC: supplementary motor cortex; SPL: superior parietal lobule.

**
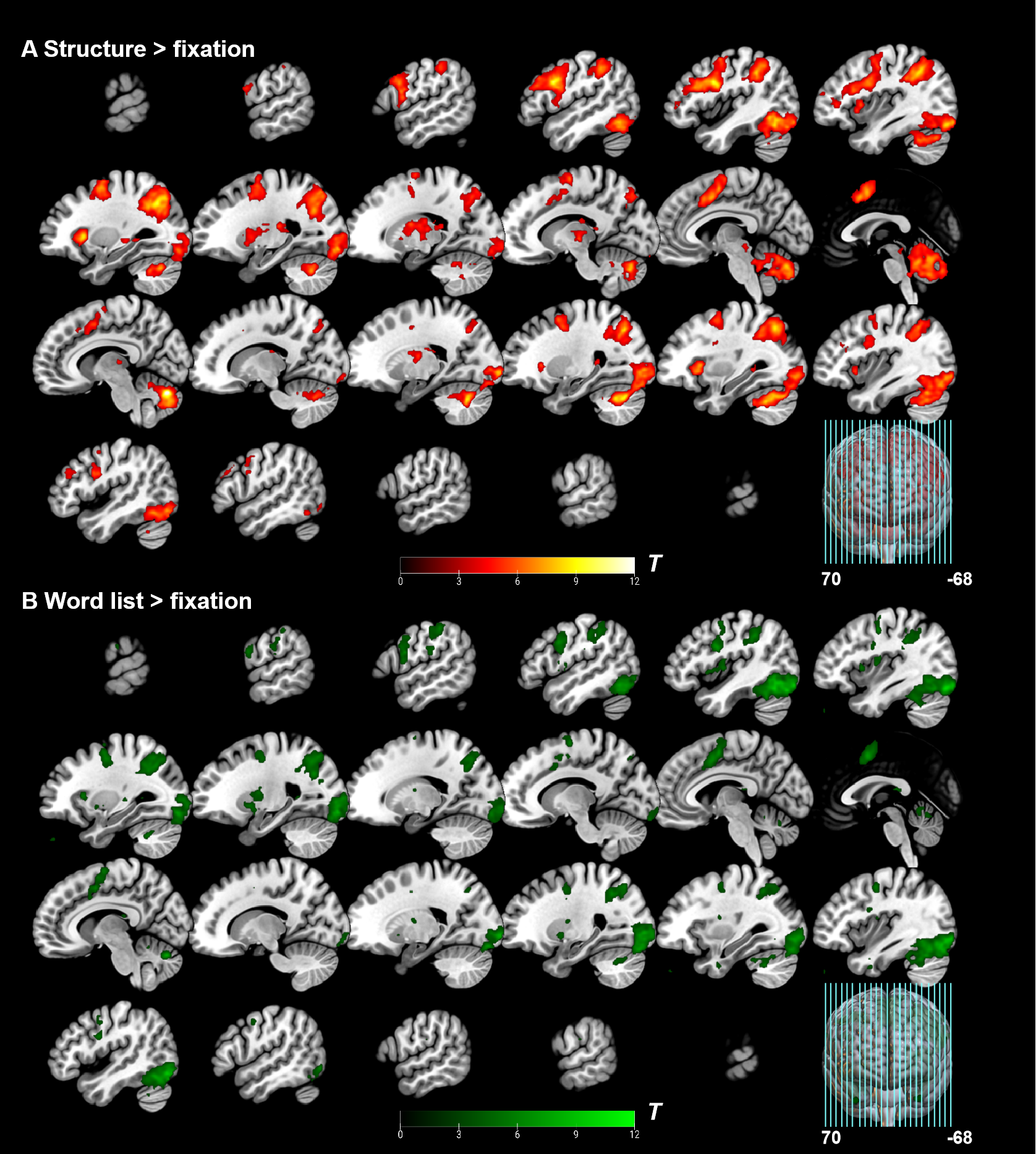
Figure S2.** Whole-brain level activation results for each condition. Upper-panel: Results under the contrast of “structure > implicit baseline (fixation)”; Lower-panel: Results under the contrast of “word list > implicit baseline (fixation)”. The render view was presented from left to right hemisphere. Activation thresholds: cluster-level: *p_FWE_* <.05, voxel-level (cluster-defining): *p_uncorr_* < .001, *K_E_* ≥ 20.

# “Session 1 > Session 2” vs. “structure > word list” at the second session

Individual contrast results for “structure > word list” were included for the second-level analysis. At the whole-brain level, paired-samples t-tests were performed to evaluate the activation differences between Session 1 and Session 2 during the scanning phase. Whole-brain results showed that compared with Session 2, Session 1 mainly induced greater activation of the bilateral insula, left parietal operculum, and right lingual gyrus (Table S2 and Figure S3) and that no supra-threshold activation could be detected for the reverse contrast of “Session 2 > Session 1”. This finding might be explained as follows: participants gradually adapted to the task of the structure condition for hierarchical phrase building, and in the first session, they spent more effort on this task (see also Zaccarella et al., 2015), which is also in line with the behavioral performance changes (see the *Main Text*). However, in the second session, although the performance difference between “structure” and “word list” was comparable to the performance difference between the two sessions, the contrast of “structure > word list” still revealed significant activation of the left IFG (mainly overlapping with Broca’s area, see Table S2 and Figure S3). Therefore, the activated region/cluster within Broca’s area only selectively responds to syntactic processing instead of being an executive control region that generally responds to task demands (or cognitive loads).

**Table S2. Activation results**

| **Contrast** | **Region** | ***K_E_*** | **MNI Peak Coordinates (mm)** | | | ***T*-value** |
| --- | --- | --- | --- | --- | --- | --- |
|  |  |  | **x** | **y** | **z** |  |
|  | | | | | | |
| **Session 1 > Session 2** | Left Ins | 143 | -30 | 20 | -14 | 9.90 |
|  | Right Ins | 95 | 30 | 20 | -14 | 7.57 |
|  | Left PO | 70 | -52 | -32 | 18 | 6.53 |
|  | Right LG | 141 | 6 | -78 | -10 | 5.81 |
| **Structure > Word List** (at the second session) | Left IFG | 867 | -48 | 28 | 22 | 8.93 |
|  | Right MFG | 195 | 30 | 10 | 52 | 6.94 |
|  |  | 163 | 46 | 30 | 26 | 6.57 |
|  | Left SMC | 173 | -4 | 10 | 40 | 6.53 |
|  | Left MFG | 277 | -28 | 0 | 54 | 6.20 |
|  | Right ITG | 22 | 52 | -46 | -18 | 5.07 |


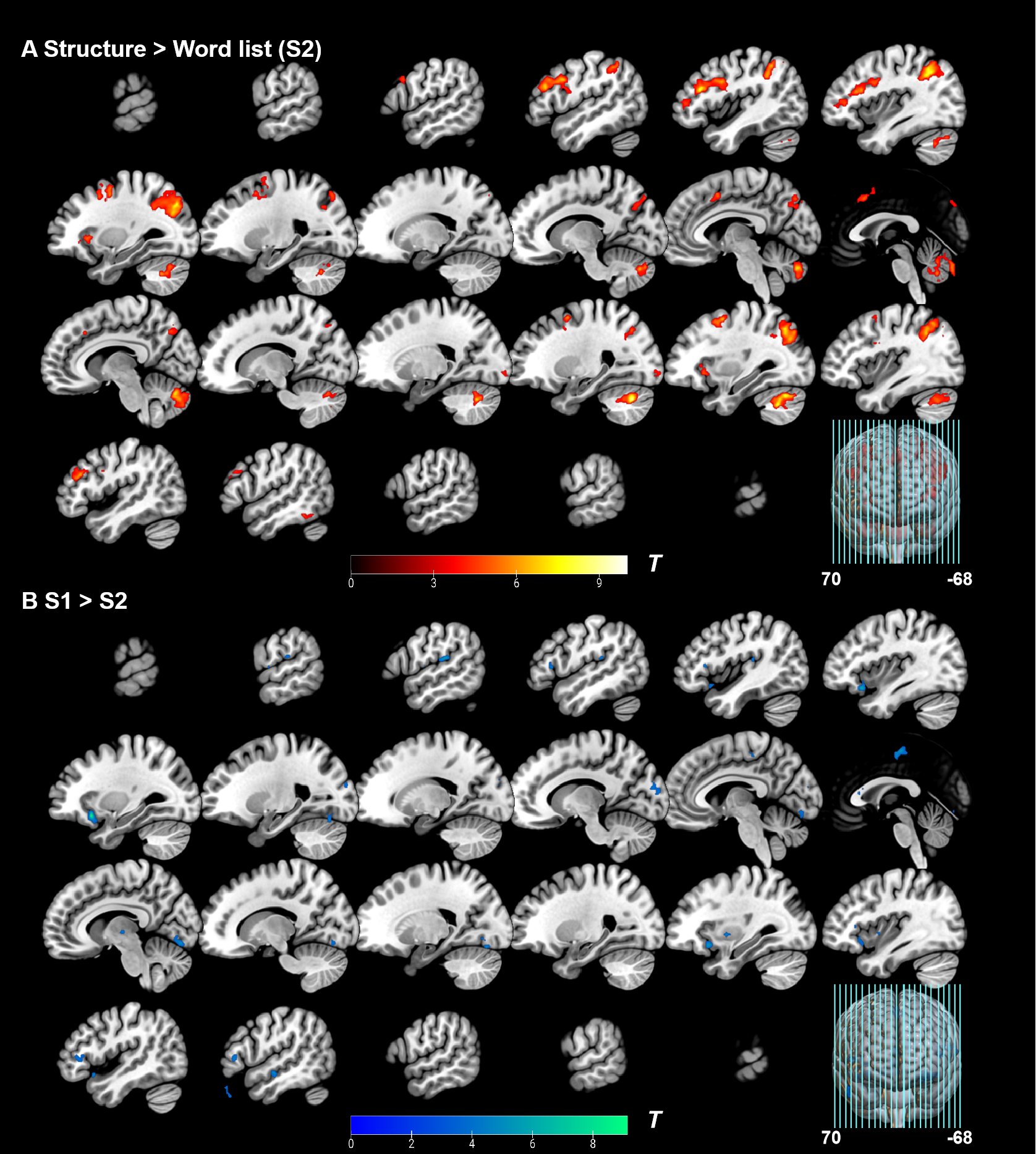
***Notes.*** Whole-brain level activation results under the contrast of “Session 1 > Session 2” and under the the contrast of “Structure > Word List (at the second session)”. Activation thresholds: cluster-level: *p_FWE_* <.05, voxel-level (cluster-defining): *p_uncorr_* < .001, *K_E_* ≥ 20. Only clusters with % white matter < 1/3 were reported. Abbreviations: Ins: insula; PO: parietal operculum; LG: lingual gyrus; ITL: inferior temporal gyrus; IFG: inferior frontal gyrus; MFG: middle frontal gyrus; SMC: supplementary motor cortex. Activation results of cerebellum were of no interest and not reported here.

**Figure S3.** Whole-brain level activation results under the contrast of “Structure > Word List (at the second session, S2)” (A) and under the contrast of “Session 1 (S1) > Session 2 (S2)” (B). Activation thresholds: cluster-level: *p_FWE_* <.05, voxel-level (cluster-defining): *p_uncorr_* < .001, *K_E_* ≥ 20. Only clusters with % white matter < 1/3 were reported.

# Comparisons between different ROIs

The functional localization approach adopted at the individual-level ROI analyses could provide us with peak activity coordinates with more statistic power under the contrast of “structure > word list”. As shown in Figure S4, signal intensities (i.e., signal change%) of the ROIs defined by the group-level ROI analyses were sparsely and inconsistently correlated with the behavioral performances. In contrast, signal intensities of the functional ROIs defined by the individual-level ROI analyses could predict the behavioral performances sensitively. These results demonstrated the necessity of resorting to the functional localization approach to improve the statistic power for ROI analyses in the present study.


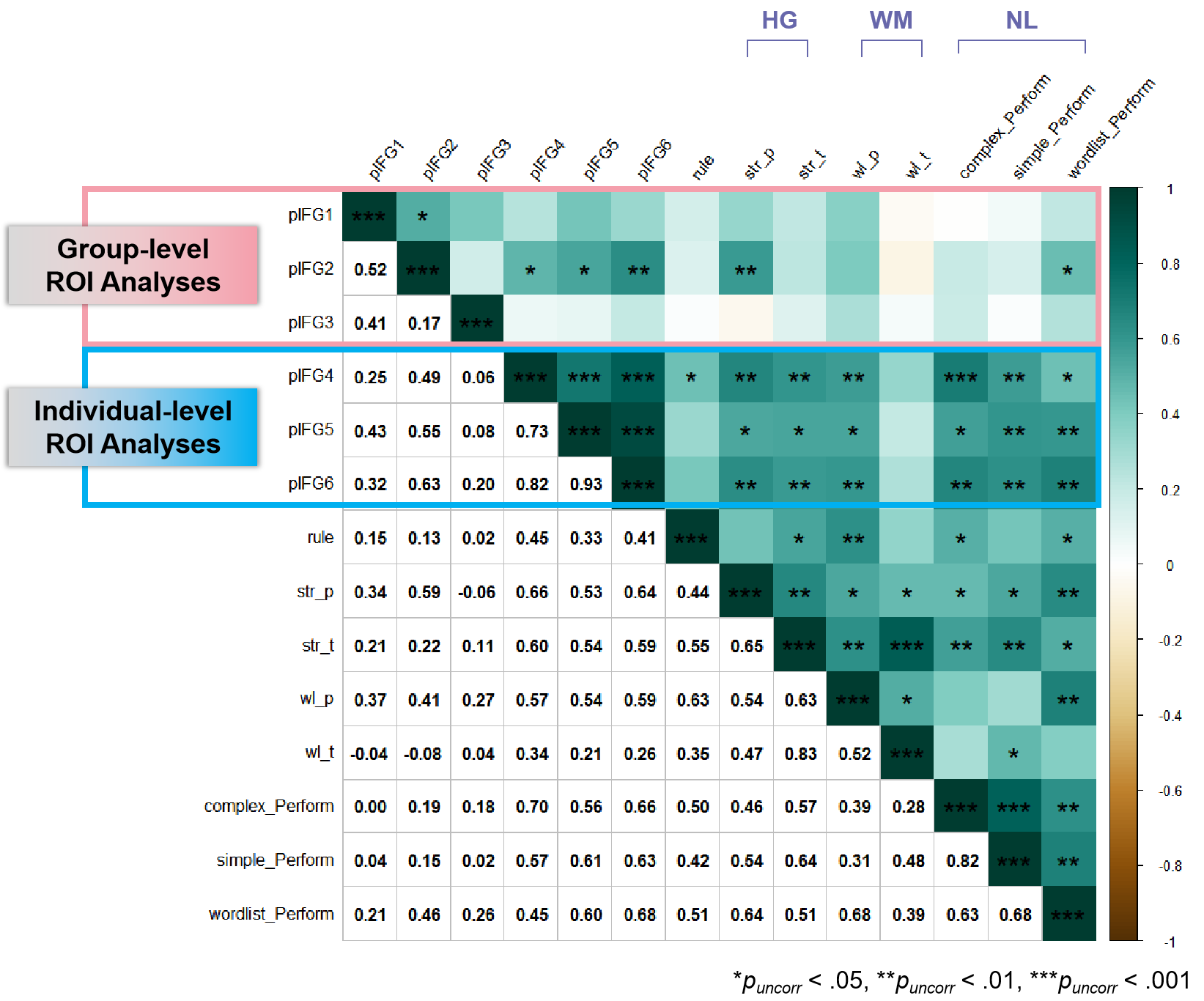
Figure S4. Comparisons of the “signal change% and behavioral performances” correlations between the individual- and group-level defined ROIs. The pIFG1-3 were defined by the group-level ROI analyses: pIFG1: within the IFG of the language atlas, x = -50, y = 18, and z = 28; pIFG2: within Broca’s area, x = -50, y = 14, and z = 30; pIFG3: averaged peak activity coordinates of pIFG1and pIFG2, x = -50, y = 16, and z = 29. The pIFG4-6 were defined by the individual-level ROI analyses: pIFG4: functional localization without picking up the top 10% localizer-responsive voxels, x = -52, y = 12, and z = 32; pIFG5: functional localization with picking up the top 10% localizer-responsive voxels, x = -54, y = 14, and z = 30; pIFG6: averaged peak activity coordinates of pIFG4and pIFG5, x = -53, y = 13, and z = 31. To note, centered on these coordinates, 4-mm-radius spheres were built up as the ROIs.

# SI References

Zaccarella, E., and Friederici, A.D. (2015). Merge in the Human Brain: A Sub-Region Based Functional Investigation in the Left Pars Opercularis. *Frontiers in Psychology* 6. doi: ARTN 01818 10.3389/fpsyg.2015.01818.
